# Small Organic Molecule-Based Multicolor Fluorescent Smart Materials: From Design Principles and Optical Properties to Versatile Applications

**DOI:** 10.1021/acscentsci.6c00693

**Published:** 2026-05-18

**Authors:** Zhen Yan, Taihong Liu, Liping Ding, Yu Fang

**Affiliations:** Key Laboratory of Applied Surface and Colloid Chemistry of Ministry of Education, Shaanxi Provincial Key Laboratory of New Concept Sensors and Molecular Materials, School of Chemistry and Chemical Engineering, 12401 Shaanxi Normal University, Xi’an 710119, P. R. China

## Abstract

As an emerging frontier in functional material science, multicolor fluorescent smart materials have garnered significant interest because of their inherent versatility and precisely tunable fluorescent characteristics, enabling the development of increasingly sophisticated and functional platforms. In this Outlook, we summarize the recent advances in small-organic-molecule-based multicolor fluorescent materials, covering their design principles, optical properties, and versatile applications. The various strategies for achieving multicolor fluorescence emission could range from classic approaches based on modulation of electronic excited states and molecular intrinsic structure to newly emerging methods, such as molecular conformation switching and regulation of molecular aggregation/packing. We also present the advanced applications of the multicolor fluorescent materials driven by these design strategies, including fluorescence sensing, anticounterfeiting, information encryption, and decryption. Finally, an outlook on the main challenges and future opportunities for multicolor fluorescent materials is previewed, aiming to accelerate the advancement of smart materials and devices. We hope that these insights will inspire further innovative research on multicolor fluorescent smart materials and their advanced applications.

## Graphical abstract

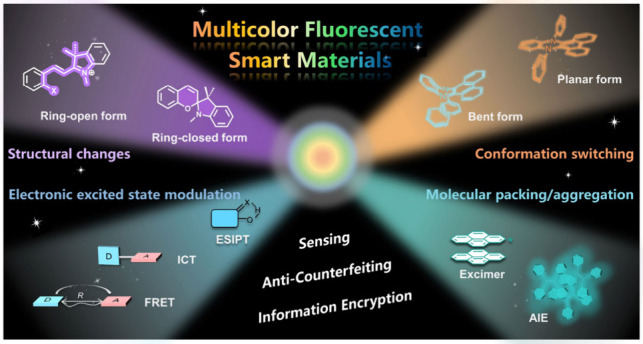

## 1 Introduction

Smart materials, also referred to as stimulus-responsive materials, exhibit reversible and visible responses when subjected to various external stimuli, such as chemicals, pH, mechanical force, temperature, light, and humidity.^1–3^ They have attracted intense and broad interest for their exceptionally versatile applications.^4–6^ Early intelligent materials were mostly based on “on–off” or “color–colorless” switching, with a single information dimension. As an important branch of stimulus-responsive materials, multicolor fluorescent smart materials exhibit a dynamically tunable fluorescence emission intensity or ratio in response to external stimuli, typically outputting more complex and diverse emission signals or colors.^7–9^ The unique combination of stimulus responsiveness and multicolor fluorescence provides a powerful visual signaling tool for applications such as chemical sensing and information encryption.^10–12^ Therefore, extensive research studies have focused on achieving precise, dynamic, and controllable regulation of the color, intensity, and lifetime of multicolor fluorescent smart materials, and endowing them with high sensitivity, fast response, and excellent reversibility to external stimuli.^13–17^

It is worth noting that the properties of fluorescent materials, whether static luminescence or dynamic responsiveness, are intimately linked to their physical states (solution, aggregate, solid).^18,19^ From the free movement of molecules in solution to ordered/disordered aggregation and highly ordered lattice structures, phase transitions profoundly affect the local environment of the luminescent center, energy-transfer processes, excited-state dynamic paths, and sensitivity to external stimuli.^20–22^ Therefore, the rational design of fluorescent molecules and deep understanding of their photophysical properties under different circumstances remain critical steps in constructing high-performance stimuli-responsive materials.^23–26^

This Outlook explores recent advances in multicolor fluorescent smart materials, detailing design strategies and stimulus-responsive behaviors. Up to now, a variety of important materials have been employed to explore multicolor emission and applications. For example, rare-earth-doped materials are widely used as they offer unique advantages in terms of color purity and fluorescence lifetime. However, their color tuning is highly dependent on precise host-lattice engineering and specific dopant combination. Carbon-based nanomaterials are admired for their high biocompatibility and facile synthesis. Their challenges lie in the heterogeneity of the emission sites. Supramolecular luminescent materials typically involve specific assembly and interactions between different components (e.g., cyclodextrin and fluorophore, cucurbituril, and fluorophore). As many excellent reviews have introduced the advancements of multicolor materials based on rare-earth-doped fluorescent materials,^27^ supramolecular luminescent materials,^28,29^ and carbon-based nanomaterials,^30^ these materials are excluded from the scope of this paper. Herein, this Outlook highlights the development of small-organic-molecule-based multicolor fluorescent smart materials, with a focus on the well-defined structure and underlying photophysical mechanisms, design strategies, multicolor fluorescence regulation, and their potential applications. We first outline strategies rooted in electronic state regulation and intrinsic molecular structure changing and then delve into emerging approaches that exploit conformation variation and molecular packing/aggregation to manipulate multicolor systems. The properties of these materials are summarized alongside a critical analysis of current strategic limitations. Crucially, an outlook into the main challenges confronting this field and potential future opportunities is previewed, anticipating the advancement of the development of multicolor smart materials and devices.

## 2 Design Strategies and Overview of Developments

Generally, the multicolor emission property of organic molecules arises from two or more emissive excited states. Thus, the generation of multicolor emission is intimately linked to the unique molecular structure and intricate photophysical competition processes within organic molecules. Therefore, a variety of strategies through modulating the electronic excited states, molecular structure/conformation, or varying the molecular packing/aggregation state have been applied to develop multicolor fluorescent materials.

### 2.1 Electronic Excited State Modulation

Modulating the electronic excited state of fluorophores is an effective tool for obtaining multicolor fluorescent materials. Precise molecular design enables dynamic control over luminescence color and efficiency by regulating the energy, lifetime, energy-transfer paths, and radiative/nonradiative transition rates of the excited states. The main luminescence conversion mechanisms include intramolecular charge transfer (ICT), excited-state intramolecular proton transfer (ESIPT), and fluorescence resonance energy transfer (FRET), which can provide different emission paths and result in multiple emissions.^31–33^

Modulating the electronic excited state of fluorophores is an effective tool to obtain multicolor fluorescent materials.

The ICT effect involves electron redistribution between a donor (D) and an acceptor (A) linked by a π-conjugated bridge in an organic molecule. Photoexcitation induces electron transfer from D to A via the π-linker, producing a charge-separated excited state (Figure 1a). The large dipole moment difference between this excited state and the ground state makes the fluorescence emission highly sensitive to the microenvironment.^33,34^ Luminescence properties based on the ICT process can be modulated through three ways: (1) adjusting conjugation degrees by extending or shortening the π-linker; (2) modulating D/A strength through tailored electron-donating or withdrawing capabilities; and (3) enhancing molecular rigidity to impose structural constraints that suppress nonradiative decay paths. By reducing the LUMO energy level or increasing the HOMO energy level through adjusting electron-donating and electron-accepting moieties, fluorescent materials with longer absorption and emission wavelengths can be obtained and display distinct solvato-fluorochromism properties.^35–37^ Such properties make asymmetric thiazolothiazole dyes color-variable and highly suitable for applications in polarity and temperature sensing, with Stokes shifts ranging from 450 to 565 nm (Figure 1d). Furthermore, the ICT process is closely related to the molecular conformation, particularly notable for the twisted intramolecular charge transfer (TICT) process. The TICT process occurs in molecular systems having a large rotational freedom between the D and A units.^38^ Upon photoexcitation, there is a torsion between the D and A units, and an almost vertical relaxation structure is generated. The balance between the twisted conformation and the coplanar conformation leads to a related dual fluorescence emission property, namely, the emission of the localized excited state at the higher energy state, and the TICT state emission at the lower energy state. Thus, it enables TICT molecules with multiple color emission properties.^39–41^

**Figure 1:**
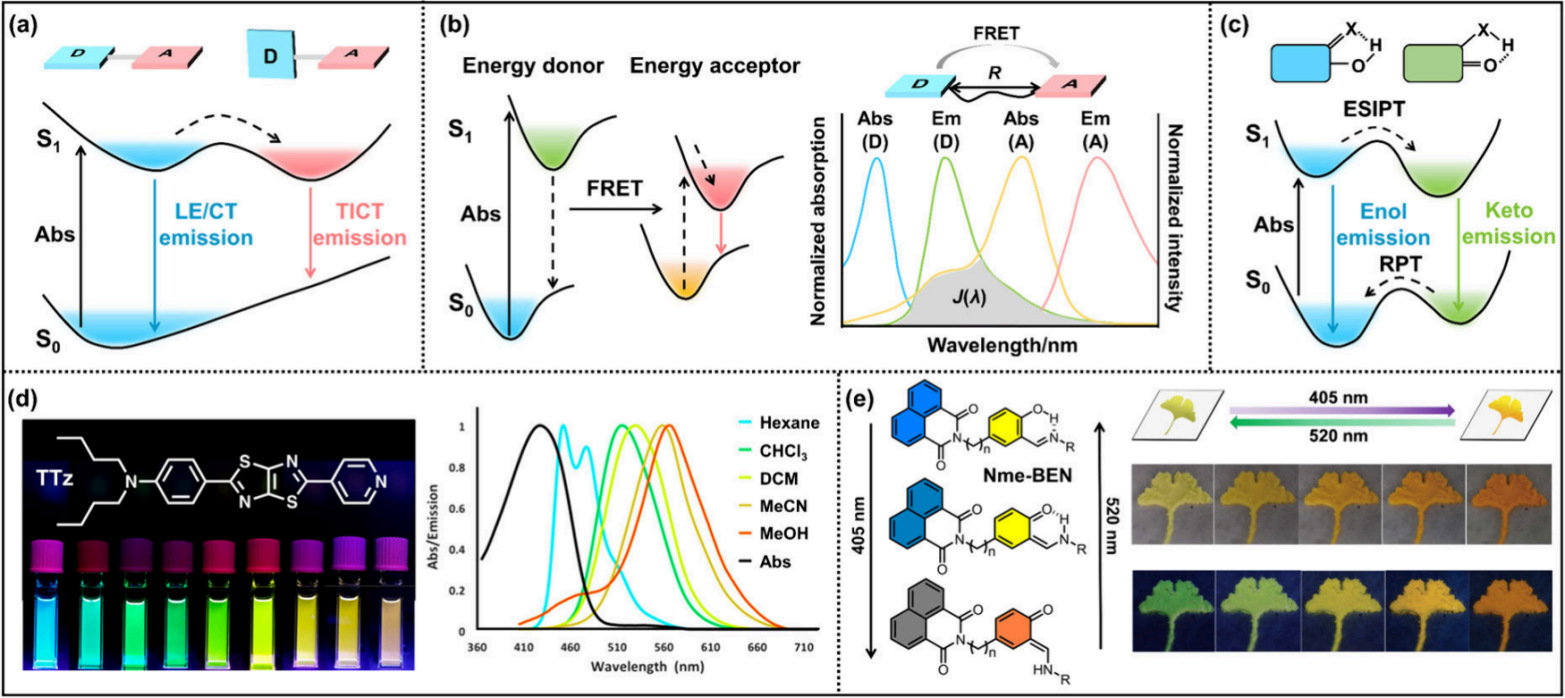
Jablonski diagrams that illustrate different photophysical mechanisms: (a) intramolecular charge transfer (ICT);^9^ (b) fluorescence resonance energy transfer (FRET);^42^ (c) excited state intramolecular proton transfer (ESIPT);^48^ (d) structure of thiazolothiazole dye and the solvent-dependent fluorescence property (solvents from left to right: hexane, toluene, Cl-benzene, CHCl_3_, ethyl acetate, CH_2_Cl_2_, acetone, ethanol, methanol);^37^ (e) Nme-BEN structure and reversible color change of Nme-BEN powder upon irradiation.^50^ S_0_ represents the ground state, and S_1_ the first singlet excited state. Abbreviations: Abs for absorption, LE for locally excited state, CT for charge transfer, TICT for twisted intramolecular charge transfer, Em for emission, *J*(λ) expresses the degree of spectral overlap between the donor emission and the acceptor absorption; RPT for reverse proton transfer, R for the distance between the donor and acceptor, FL for fluorescence. Panel (a) reproduced with permission from ref 9. Copyright 2025 Wiley-VCH. Panel (b) reproduced with permission from ref 42. Copyright 2013 American Chemical Society. Panel (c) reproduced with permission from ref 48. Copyright 2021 Wiley-VCH. Panel (d) reproduced with permission from ref 37. Copyright 2019 American Chemical Society. Panel (e) reproduced with permission from ref 50. Copyright 2024 Wiley-VCH.

FRET is a nonradiative process whereby the excited-state energy from a D fluorophore transfers to a nearby A fluorophore via dipole–dipole interactions, enabling subsequent radiative emission from the A unit (Figure 1b). Effective FRET systems require three fundamental conditions, including spatial proximity (<10 nm) between D and A, significant spectral overlap between D emission and A absorption spectra, and favorable dipole alignment orientation.^22,42^ Commonly employed donors include 1,8-naphthalimide, pyrene, coumarin, and tetraphenylethylene (TPE), while representative acceptors encompass rhodamine, spiropyran, Nile Red, and fluorescein derivatives.^43–45^ The adjustable FRET efficiency and the combination of donor and acceptor emission could lead to multicolor emission.^46^

Besides charge transfer and energy transfer, proton transfer in a fluorophore can also be used to produce variable multicolor emission. In a typical ESIPT process, the photoexcitation triggers intramolecular hydrogen bonding (e.g., -OH/-NH_2_ with C=O/N/S), facilitating proton transfer and reversible enol-keto tautomerization between the ground and excited states (Figure 1c). This process produces distinct photophysical changes, a large Stokes shift, and dual fluorescence emission, which provides the possibility of adjusting multicolor emission. Common ESIPT units include benzazole derivatives, hydroxyflavone derivatives, salicylaldehyde derivatives, hydroxyquinoline derivatives, and orthohydroxyacetophenone derivatives.^47–49^ The combination of ESIPT and *cis*-*trans* isomerization can generate a photoresponsive material (Figure 1e). The representative molecule Nme-BEN exhibits a distinct emission peak (553 nm) attributed to the *cis*-keto form and a gradual enhancement of the *trans*-keto emission at 582 nm via *cis*-to-*trans* isomerization.^50^ The associated color change of Nme-BEN powder could be applied to mimic natural development of a maple leaf from initial green to yellow and then to deep orange.

Collectively, electronic excited-state modulation provides a solid theoretical and technical foundation for the production of multicolor fluorescent materials. This not only further enriches molecular design approaches but also broadens the scope and modes of stimulus responses, yielding smart materials with an excellent tunability and predictability.

### 2.2 Intrinsic Molecular Structural Changes

Molecular structural changes act as the basic trigger for fluorescence-color switching. Introducing responsive groups is one of the most common ways to realize the structural changes. Representative responsive groups include spirolactam,^51^ spiropyran,^52^ azobenzene,^53^ and amines,^54^ which are capable of performing reversible structural changes through *cis/trans* isomerization, ring-opening/closing isomerization, and protonation/deprotonation processes upon external stimuli. The switchable structures endow these responsive groups with different electronic distribution states and conjugation degrees, thereby altering their photophysical properties, such as absorption/emission wavelength and intensity. Clearly, organic fluorophores with these functional groups can be used to develop multicolor materials, as they are capable of performing reversible molecular structural changes.^55,56^

Molecular structural changes act as the basic trigger for fluorescence color switching.

Compounds including α-cyanostilbenes,^57^ hydrazones,^58^ azobenzenes, and azopyrazoles^53,59^ undergo *cis*/*trans* isomerization upon light irradiation, both in solution and solid states (Figure 2a-1).^55^ This photoinduced isomerization is often accompanied by a significant shift in the wavelength of fluorescence emission, producing visible blue-shifted or red-shifted emission. The fluorophores exhibiting rapid isomerization and fluorescence variation can be used to generate rewritable optical patterns, demonstrating the promising applications in advanced information storage system.^60^

**Figure 2:**
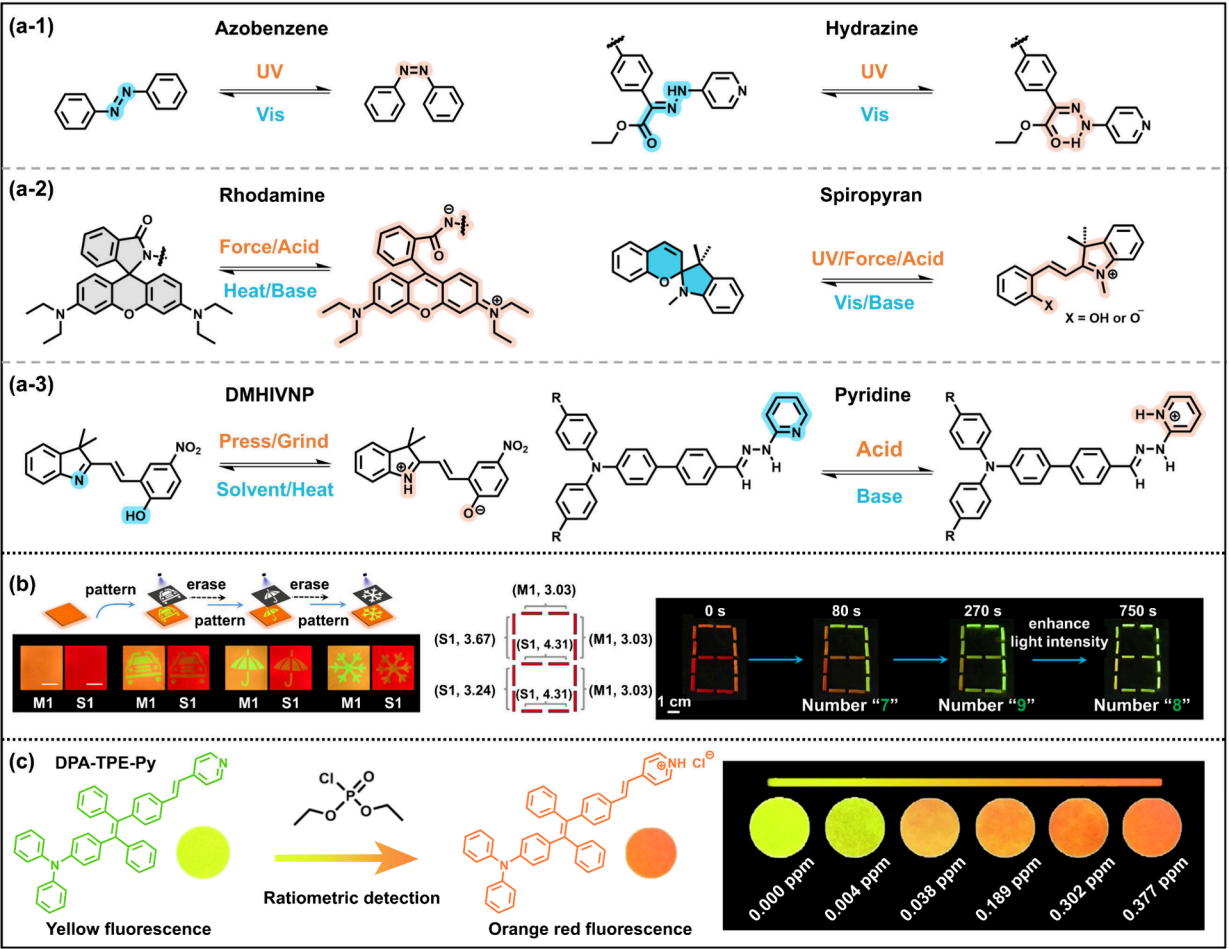
(a) Several functional groups based on molecular structure changes: (a-1) *cis/trans* isomerization; (a-2) open/closed-ring isomerization; (a-3) protonation/deprotonation process; (b) repeating the process of light patterning and dark erasing based on spiropyran structure; photochromic multicolor spiropyran hydrogels at specific pH values upon irradiation;^61^ (c) color change of DPA-TPE-Py test strips in response to diethyl chlorophosphite vapor.^62^ Panel (b) reproduced with permission from ref 61. Copyright 2025 Wiley-VCH. Panel (c) reproduced with permission from ref 62. Copyright 2016 Royal Society of Chemistry.

The reversible open/closed-ring isomerization usually involves the interconversion between a fluorescent conjugated open form and a closed form with quenched fluorescence due to broken conjugation (Figure 2a-2). A variety of spiropyran- and spirolactam-based derivatives have been reported for developing multicolor materials, which undergo ring-opening/closing switches upon external stimuli including pH,^63^ mechanical force,^64^ and acid–base titration.^52^ Such probes can be embedded into hydrogels to produce multicolor fluorescent materials with spectral coverage up to 250 nm upon light stimulus.^61^ Concurrently, the isomerization-triggered change in molecular charge could as well modulate the shape of the hydrogels. By controlling the pH, light intensity, or hydrogel thickness, the speed and synchronization of color switching and shape changes could be precisely regulated (Figure 2b).

The protonation/deprotonation process represents a fundamental strategy for molecular structural transformation. By regulating the protonation state via acid/base stimuli, the electronic structure and photophysical properties are significantly altered, enabling reversible switching of fluorescence color or intensity. These changes typically arise from altered intramolecular charge distribution, expansion/contraction of conjugated systems, or energy-level transitions induced by protonation/deprotonation. Such molecules (e.g., fluorescein, quinoline, pyridine derivatives) usually contain basic (e.g., amino groups, pyridine nitrogen) or acidic (e.g., phenolic hydroxyl groups, carboxyl groups, oxime groups) functional groups (Figure 2a-3).^54,65^ As shown in Figure 2c, upon exposure to diethyl chlorophosphite vapor, the pyridine group was converted to a pyridinium group, leading to a red shift in the emission spectrum from 546 to 624 nm.^62^

Classical strategies centered on molecular structural modification and electronic state modulation have laid a precise and robust foundation for constructing multicolor fluorescent materials while exhibiting exceptionally high designability and responsiveness. However, such systems face several limitations, including restricted structural tunability, narrow stimulus selectivity, and a constrained response mode. It is notable that stimulus-induced structural or electronic changes rarely occur independently. They are invariably accompanied by conformational transitions, changes in molecular packing, and intermolecular interactions. Consequently, emerging research frontiers are now focusing on regulating the molecular conformation, packing modes, and aggregate states, thereby paving the way for the next wave of breakthroughs in multicolor fluorescent materials.

### 2.3 Molecular Conformation Switching

Dynamic switching in molecular conformation represents a core mechanism for regulating the photophysical properties of multicolor fluorescent materials. In the excited state, subtle conformational adjustments (such as intramolecular bond angle rotations, spatial folding/unfolding, or backbone twisting) can significantly alter electronic transition paths and exciton relaxation processes. This thereby endows the materials with encoded and diverse fluorescence emission. The conformation-dependent molecules include *N*,*N*’-diphenyl-dihydrodibenzo­[*a*,*c*]­phenazine (DPAC) derivatives,^66^ phenothiazine derivatives (PTZ),^67^ and *o*-carborane compounds.^68,69^

Dynamic switching in molecular conformation represents a core mechanism for regulating the photophysical properties of multicolor fluorescent materials.

DPAC and its derivatives serve as good candidates for the fabrication of multicolor fluorescent smart materials. They possess a unique “saddle-shaped” structure, exhibiting distinctive properties such as dual fluorescence emission (a blue emission band and a red emission band), a large Stokes shift, and responsiveness to diverse environmental stimuli. This unique photophysical behavior is rationalized by vibration-induced emission (VIE), which arises from the vibrational motions of the two aromatic moieties along the N–N axis, driving excited-state conformational changes from a bent (blue-emitting) to a planar (red-emitting) configuration and thereby enabling multicolor fluorescence emission (Figure 3a).^70,71^ A variety of environmental factors can modulate the conformational changes of DPAC derivatives, such as polarity, viscosity, and temperature. Thus, DPAC derivatives have been widely applied for potential fields such as solvent discrimination,^72^ temperature sensing,^73^ biological sensing,^3^ and optical display.^74^ As depicted in Figure 3b, viscosity restricted the intramolecular vibrations of DPAC, resulting in the bent form with blue emission.^75^ The emission color changes were visualized from red to blue via white light emission, with the emission peaks shifting by 155 nm.

**Figure 3:**
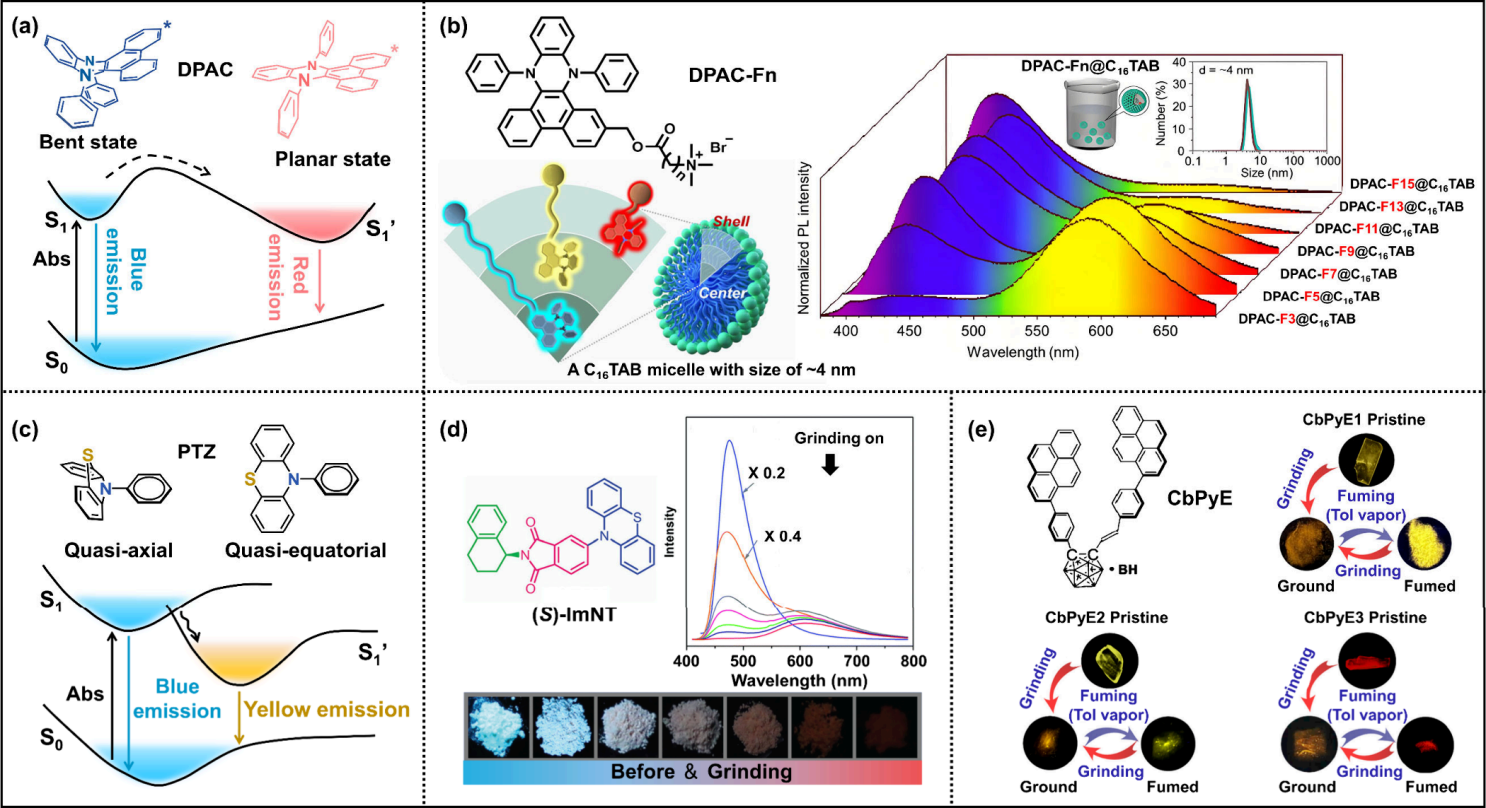
(a) Structure of DPAC and the mechanism of vibration-induced emission;^70^ (b) structure of DPAC-Fn and the corresponding emission spectra of DPAC-Fn@C_16_TAB in aqueous media;^75^ (c) structure of PTZ and fluorescence-conformation process; (d) variations in emission properties of *S*-ImNT triggered by external force;^78^ (e) zigzag structure of CbPyE and the fluorescence responses of various CbPyE crystals upon grinding and exposure to toluene vapor.^80^ Panel (a) reproduced with permission from ref 70. Copyright 2020 Wiley-VCH. Panel (b) reproduced with permission from ref 75. Copyright 2023 American Chemical Society. Panel (d) reproduced with permission from ref 78. Copyright 2019 Royal Society of Chemistry. Panel (e) reproduced with permission from ref 80. Copyright 2024 American Chemical Society.

PTZ derivatives, benefiting from a distinctive “butterfly-like” spatial conformation and electron-rich property, exhibit excellent smart fluorescent behaviors.^76^ PTZ can undergo folding along the N–S axis, which confers a balance of rigidity and flexibility. The conformational change alters the electron-donating ability of the PTZ unit, and consequently induces different emission behaviors (Figure 3c). It effectively suppresses intermolecular π-π stacking interactions, thereby enhancing the fluorescence efficiency of PTZ molecules in the aggregate state.^67^ Quasi-axial and quasi-equatorial conformations readily interconvert upon mechanical stimulation (grinding, pressure). This conformation change can directly cause a change in the aggregates (molecular packing modes), accompanied by significant differences in emission color.^77^ The multicolor fluorescence of a PTZ derivative has been witnessed through a single mechanical stimulus, where grinding leads to vanishing of the blue emission at 478 nm and the generation of red emission at 618 nm (Figure 3d).^78^ The large spectral shift of up to 140 nm enables the material to be tuned to even emit white light.

The *o*-carborane possesses a unique polyhedral cage-like structure, featuring a highly symmetrical and rigid three-dimensional structure. Coupled with the significant difference in electronegativity between carbon and boron atoms, the *o*-carborane becomes a distinctive electron acceptor.^79^ Its rigid cage-like structure effectively inhibits close molecular packing, helping to maintain the luminescence efficiency in the aggregation state. More importantly, the highly reactive carbon atoms at the vertices of the *o*-carborane provide key sites for molecular functionalization. Introducing different aromatic fluorescent groups (such as carbazole and pyrene) at the *o*-carborane vertices enables the construction of luminescent skeletons with significant charge transfer characteristics.^12,69^ Additionally, introducing intramolecular rotatable C–C bonds, alkyl chains, or π-conjugated structures onto the rigid *o*-carborane skeleton can facilitate the formation of highly ordered single crystals. This strategy opens an important avenue for the development of novel stimulus-responsive fluorescent materials. Single crystals derived from *o*-carborane derivatives with multiple conformations exhibit tunable yellow-to-red fluorescence changes upon grinding or vapor exposure (Figure 3e).^80^ The combination of two *o*-carborane groups with DPAC also affords a single-molecule material exhibiting excitation-dependent multicolor fluorescence. This behavior originates from the conformational flexibility of the DPAC linker, the distinctive spatial conformation of *o*-carborane, and the excimer formation via the strong intermolecular interactions between DPAC segments.^81^

As exemplified by flexible moieties (DPAC, PTZ) and rigid frameworks (*o*-carborane), molecular conformation switching offers a novel and straightforward perspective for deciphering the evolution of excited states. This strategy serves as a potential tool to endow materials with distinct multicolor fluorescence properties along with continuous and reversible stimulus-responsive behaviors. These nonplanar conjugated structures also can introduce rotatable C–C bonds or large conjugated structures within the molecule, thereby regulating the molecular conformation and affecting the packing mode and excited states. This lays the theoretical foundation for the development of a new generation of multicolor smart materials with temperature-, force-, and solvent-sensitive properties.^82,83^ The conformation-switching materials present a clear functional hierarchy. The immediate challenge lies in quantitatively elucidating aggregate conformational dynamics by integrating in situ spectroscopy with theoretical modeling. Subsequently, the current focus is on constructing multicolor smart materials with a predictable and sequential switching behavior. A major frontier is integrating these molecular switches into macroscopic devices and soft materials with desirable performance. Meanwhile, the utilization of artificial intelligence and machine-learning approaches has become indispensable for efficient and rational molecular design.

### 2.4 Molecular Packing/Aggregation

Molecular packing/aggregation serves as a crucial link connecting molecular structure/conformation with macroscopic fluorescent properties.^84^ It is driven by various noncovalent molecular interactions, giving these materials dynamic and reversible tunable fluorescence properties. The properties of the obtained materials have good predictability due to the introduction of packing groups and aggregation structure via molecular design. More and more cutting-edge research is focused on constructing multicolor fluorescent materials through molecular aggregation/packing methods.^85,86^ By precisely regulating packing modes (H-/J- aggregation), intermolecular interactions (π-π interaction, hydrogen bond, halogen bond), and the resulting excited state properties (CT state, excimers), it is possible to achieve adjustment over fluorescent color, efficiency, lifetime, and responsiveness.

Since Tang’s team first proposed the concept of “aggregation-induced emission (AIE)” in 2001, the research field related to AIE has been booming.^87^ The AIE effect refers to the phenomenon where molecules do not emit fluorescence in dilute solutions but exhibit strong fluorescence emission in the aggregated state. The essence lies in that the molecular aggregates limit the vibrational and rotational processes within the molecule that dissipate the excitation energy nonradiatively, thereby raising the barrier at the intersection point of the lowest singlet excited state S_1_ and the ground state S_0_ (Figure 4a).^88^ Restricting intramolecular motion through molecular structure, molecular packing, and molecular conformation has been proposed to elucidate AIE behaviors.^89,90^ For further reading, we recommend the recent systematic review on the stimuli-responsive AIEgens reported by Tang et al. in 2021.^91^ The AIE effect serves as a potent enabler, resolving the fundamental efficiency challenges encountered by luminescent materials during practical application, particularly in aggregated states. Building upon this foundation, flexible molecular engineering strategies have enabled full-color fluorescence tuning from deep blue to near-infrared.

**Figure 4:**
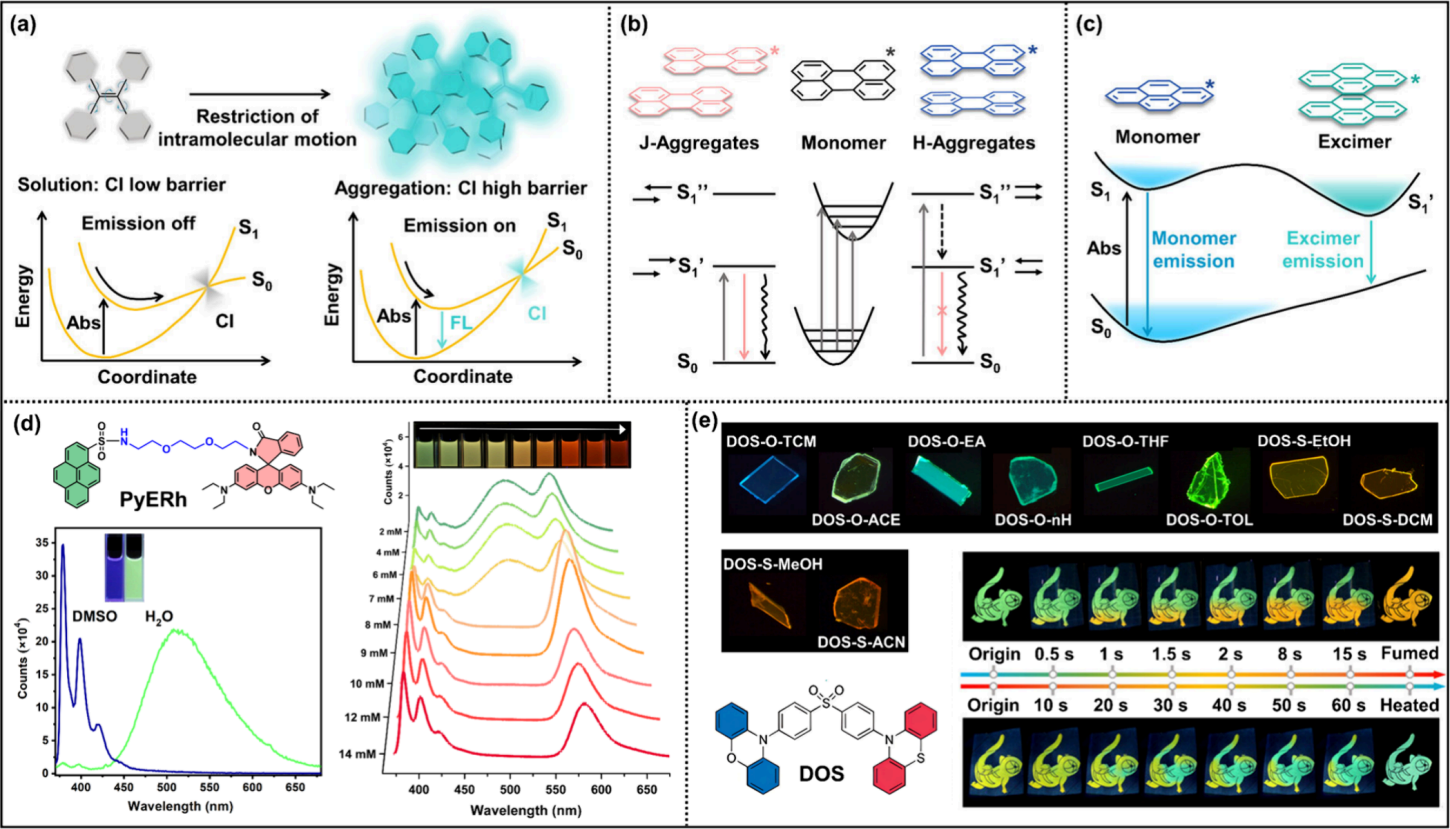
(a) Schematic mechanisms of the AIE phenomenon, CI for conical intersection;^90^ (b) H- and J-aggregates and their corresponding photophysical processes;^92^ (c) mechanism of excimer formation;^9^ (d) molecular structure of PyERh and multicolor fluorescence emission spectra in DMSO, H_2_O and different SDS-concentrated aqueous solutions;^100^ (e) Structure of DOS molecule and its multicolor emission behaviors.^101^ Panel (a) reproduced with permission from ref 90. Copyright 2015 American Chemical Society. Panel (b) reproduced with permission from ref 92. Copyright 2018 American Chemical Society. Panel (c) reproduced with permission from ref 9. Copyright 2025 Wiley-VCH. Panel (d) reproduced with permission from ref 100. Copyright 2021 Elsevier. Panel (e) reproduced with permission from ref 101. Copyright 2020 Wiley-VCH.

H- and J-type aggregations represent two typical molecular packing modes in the aggregated states, and their fluorescent properties are closely related to the exciton coupling between molecules (Figure 4b).^92^ H-aggregates feature a “face-to-face” (or sandwich-type) molecular packing, resulting in coupling of transition dipole moments, with a higher transition energy to the excited state, and the absorption and emission spectra shifting toward the shorter wavelength region. Differently, J-aggregates adopt a “head-to-tail” (or slipped, staircase-like) molecular packing, also giving rise to coupling of transition dipole moments, with a lower transition energy to the excited state, and the absorption and emission spectra shifting toward the longer wavelength region.^93,94^ Representative structures of H-/J-aggregates are perylene bisimides, anthracene, naphthalimide, and cyanine. By introducing amide bonds, bulky groups, or flexible side chains (e.g., long alkyl chains) onto the molecular framework, the resulting steric hindrance further modulates the aggregation and packing modes. This mechanism complements the molecular structure design, enabling direct and efficient regulation of the fluorescent color via precise control over molecular packing. Therefore, applications in the field of haptic sensing, melamine detection, and tumor therapy have been successfully realized.^95–99^

Excimers are typically formed from planar aromatic compounds that possess extensive π-conjugation, such as pyrene, naphthalene, and anthracene. The excited fluorescent groups (monomers) will form excimer complexes with the ground-state fluorescent groups, which exhibits a strong and broad spectral band without vibrational fine emission peak in the long spectral region (Figure 4c). The excimer emission of aromatic compounds and their functional applications are closely related not only to the molecular structure but also to their packing mode in the aggregated state.^84^ By regulating the monomer–excimer dual emission, these materials can serve as promising candidates for ratiometric sensing and multichannel imaging. The integration of a pyrene unit with a second fluorophore enables pyrene derivatives to not only exhibit monomer emission at 380 nm and excimer emission at 510 nm, but also display an extra emission band originating from the second fluorophore.^100^ Such a strategy empowers pyrene derivatives with excellent multicolor emission performance, which has been approved by a pyrene-rhodamine derivative with spectral spanning over 250 nm (Figure 4d).

Packing modes also play pivotal roles in determining the fluorescent behavior of crystalline materials. Polymorphism refers to the phenomenon where a single chemical compound can crystallize into two or more distinct crystalline forms. They frequently exhibit significantly different fluorescence properties, owing to variations in molecular packing (e.g., slip angles, interlayer spacings, and molecular interaction networks) or molecular conformations (e.g., torsion angles and bond angles). By precisely controlling the crystallization conditions or applying external stimuli (grinding, solvent fumigation, temperature changes) to induce crystal phase transitions, the fluorescent color, intensity, and luminescence type (fluorescence/phosphorescence) can be reversibly modulated.^102,103^ Such systems can be achieved by combining two units exhibiting different conformations. The combination of an O-containing phenoxazine unit and a S-containing PTZ unit resulted in a fluorophore with ten different single crystals, which exhibited broad emission ranging from 469 to 583 nm (Figure 4e).^101^ The polymorphism behavior was attributed to the conformational changes of the PTZ unit, resulting in alternating dominance of fluorescence emission between the rigid planar conformation of the phenoxazine unit and the folded deformation of the PTZ unit.

Precise control over molecular packing/aggregation, governed by weak intermolecular interactions (π-π stacking, C–H···π interaction, C–H···F interaction, and C–H···O interaction), endows single-component materials with dynamic multicolor fluorescence. This provides a unique opportunity to identify the intermolecular factors that drive the molecules packing into a particular aggregate and also opens up a new design pathway for developing novel stimuli-responsive multicolor fluorescent materials.

Precise control over molecular packing/aggregation, governed by weak intermolecular interactions (π-π stacking, C–H···π interaction, C–H···F interaction, and C–H···O interaction) endows single-component materials with dynamic multicolor fluorescence.

## 3 Advanced Application Prospects

Multicolor fluorescent smart materials, crafted through diverse rational designs, now enable unprecedented on-demand regulation of fluorescence behaviors and dynamic responses to environmental stimuli. These designs endow the materials with reversible multicolor switching, high-contrast signal output, and excellent environmental adaptability, enabling potential use for versatile advanced applications. This section highlights the versatile applications of multicolor fluorescent smart materials in sensing,^44,104^ anticounterfeiting,^105^ and reversible information encryption/decryption.^106^ The underlying working mechanisms are also particularly introduced.

### 3.1 Sensing

The stimulus-responsive properties and environmental sensitivity enable multicolor fluorescent materials to be widely applied in the sensing field. A variety of chemical or physical stimuli can induce chromatic alterations and fluorescence variations in these materials.^107,108^ The sensing targets range from chemicals such as proton-related pH and metal ions to physical factors such as temperature and mechanical force.

Multicolor fluorescent smart materials provide an ideal method for converting pH chemical signals to optical signals with high fidelity and anti-interference capability.^63,109^ They can function as a more powerful tool for pH detection with a wider range and higher precision. A multiple-responsive pH probe can be obtained by combining protonatable electron donors (e.g., dimethylamine moiety) and protonatable acceptors (e.g., carbonyl heterocyclic unit).^110^ The probe undergoes three protonated states in an acidic environment: protonated donor (H^+^D-A), protonated acceptor (D-AH^+^), and protonated donor and acceptor (H^+^D-AH^+^). This results in a nonmonotonic convex change in emission color as the pH decreases, shifting from yellow to blue and then to red. Such a nonmonotonic response, characterized by two opposite spectral shifts, allows for the discrimination of perishable food freshness. This design strategy integrates moieties with opposing luminescence behaviors into a single molecule, providing a rational approach for the biomimetic design of intelligent nonmonotonic multistimulus-responsive systems.

Metal ion detection has attracted widespread attention.^88,111^ Multicolor fluorophores play important roles in precisely monitoring metal ions by providing ratiometric responses and a concentration-relevant color output. A DPAC unit featuring multicolor emission, when integrated with a recognition group, can fulfill this objective. A representative DPAC-modified probe using thymine as the recognition unit has been well demonstrated.^112^ Upon addition of Hg^2+^ into the probe solution, the fluorescence color evolves from orange-red through white and finally to blue, with the emission peaks shifting by 131 nm. This is attributed to the fact that the complex with Hg^2+^ transformed the DPAC backbone from a planar to a bent conformation and enhanced the blue emission and then effectively regulated the fluorescence emission (Figure 5a**–**c).

**Figure 5:**
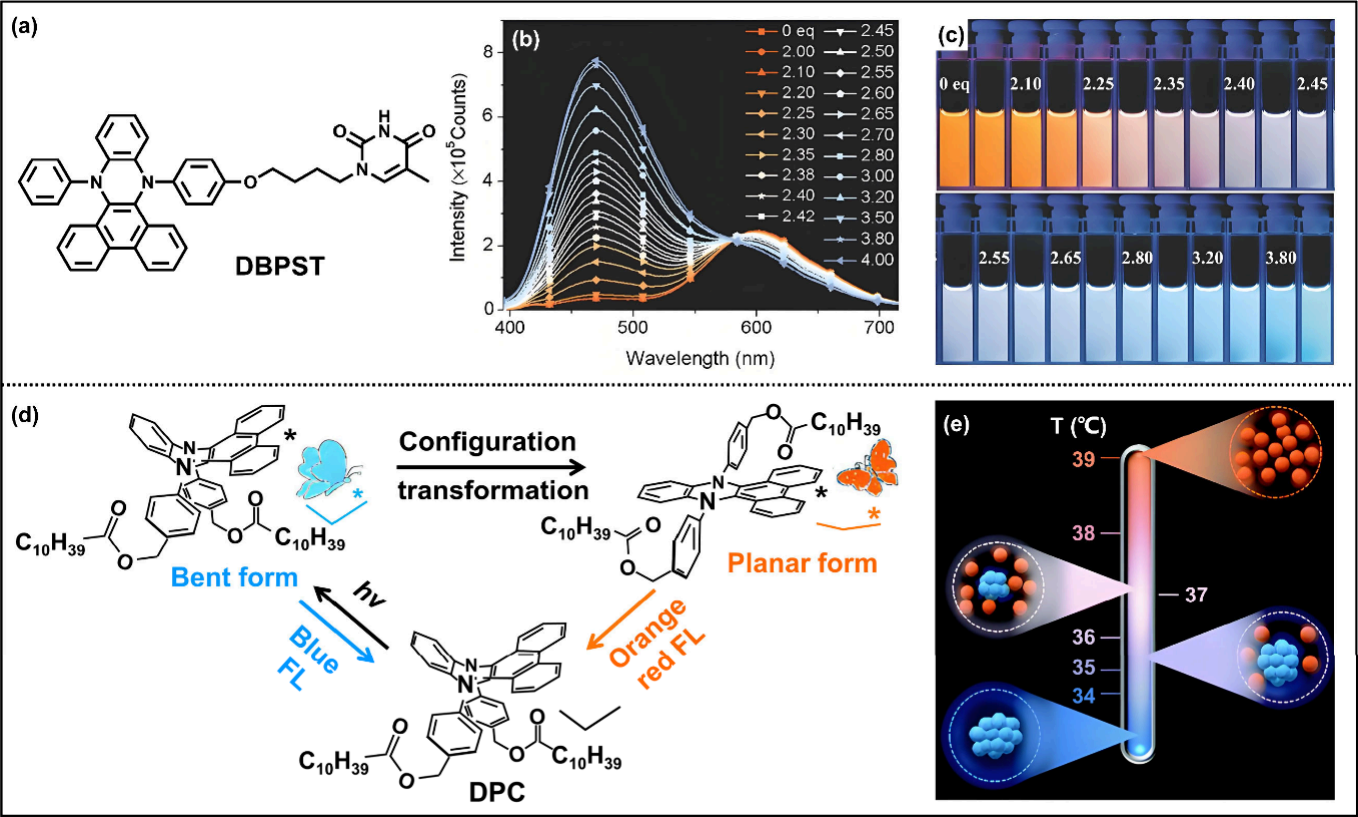
Multicolor fluorescence changes via conformation transformation: (a) structure of DBPST; (b) fluorescence spectra of DBPST with 0–4.00 equiv. Hg^2+^; (c) corresponding fluorescence photographs under 365 nm UV light illumination.^112^ (d) Illustration of the chemical structures of DPC in different conformations; (e) principle of a ratiometric thermometer utilizing aggregation-induced and disaggregation-induced transitions between bent and planar excited-state configurations of DPC.^113^ Panel (a, b, and c) reproduced with permission from ref 112. Copyright 2016 Wiley-VCH. Panel (d, e) reproduced with permission from ref 113. Copyright 2020 Royal Society of Chemistry.

Temperature sensing is widely needed in various fields. Thermochromic fluorescent smart materials demonstrate significant potential for developing next-generation fluorescent thermometers, which can provide visualized wide-range temperature detection.^114,115^ Usually, DPAC derivatives are the ideal choices for temperature-tunable emission probes. The probe exhibits different conformation-related color emissions and can be used as an ultrasensitive and ratiometric fluorescent thermometer with a broad response range (Figure 5d).^113^ Its aggregation degree in ethanol/glycerol mixtures could be precisely regulated by temperature, thus exhibiting a color variation from blue to orange in response to a temperature change from 16 to 40 °C (Figure 5e). DPAC units can be modulated through the two interconversions between the bent and planar states, rendering these derivatives promising as robust fluorescence sensing tools, and providing a feasible way to fabricate multicolor fluorescent smart materials.

A series of mechanical sensing materials based on D–A structures can be constructed by integrating force-sensitive components into caged AIE polymer chains. Upon mechanical stimulation such as shear, grind, or ultrasound applied in both solution and solid states, the unique covalent bond cleavage is triggered, generating highly tunable fluorescence from blue, green, yellow, to orange-red, with emission peaks shifting across a 138 nm range (Figure 6a).^116^ The activation of force-sensitive moieties and the regulation of energy transfer between caged and uncaged forms have opened up new prospects for the application of mechanically responsive materials in damage detection, biological imaging, and related fields.

**Figure 6:**
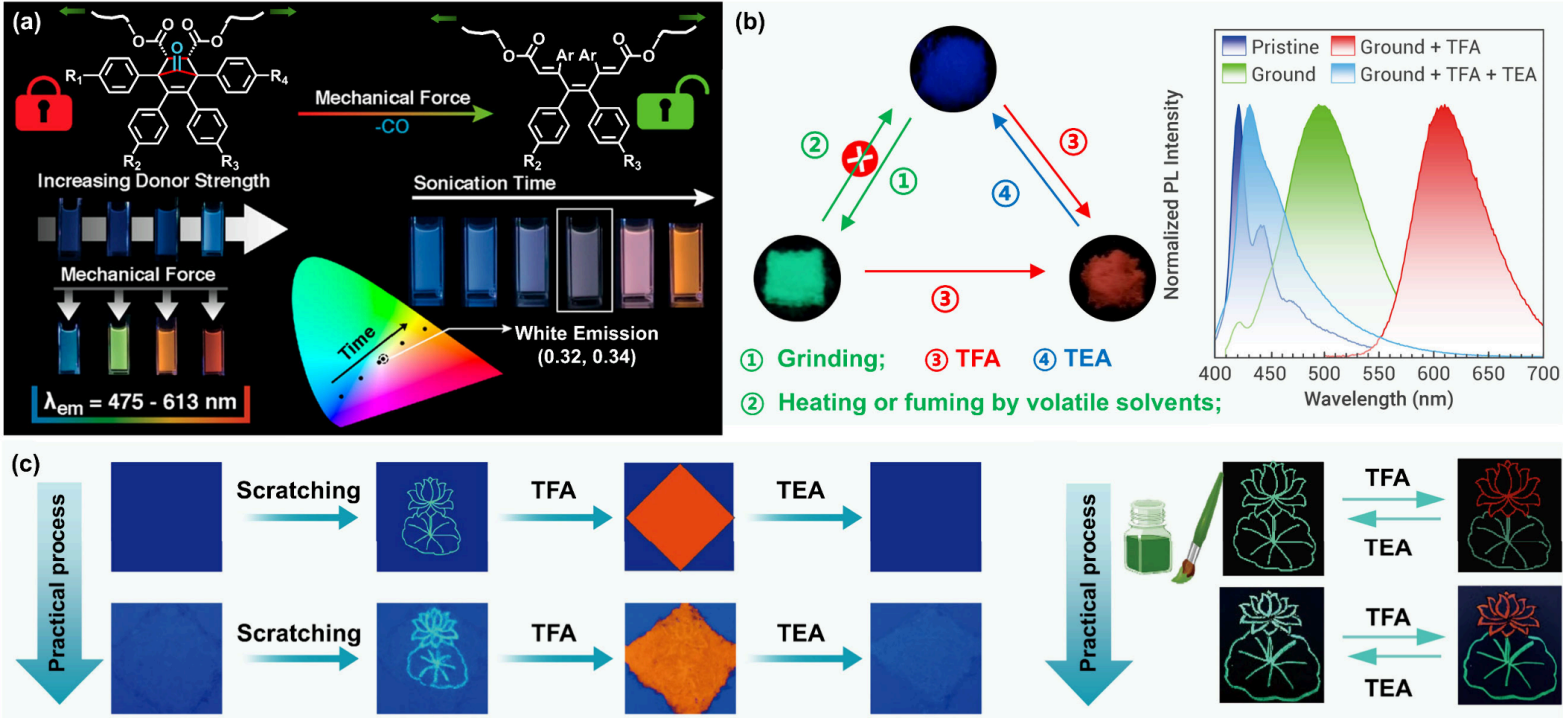
(a) Schematic illustration of the mechanically caged AIEgens with tunable emission, and the corresponding fluorescence photographs;^116^ (b) mechanochromic and acidochromic properties of Py-DAA; (c) anticounterfeiting application of Py-DAA in the solid state and solution state.^117^ Abbreviations: TFA for trifluoroacetic acid, TEA for triethylamine. Panel (a) reproduced with permission from ref 116. Copyright 2024 American Chemical Society. Panel (b, c) reproduced with permission from ref 117. Copyright 2025 Elsevier.

### 3.2 Anti-Counterfeiting

Counterfeiting issues involve economy, human health, and national security. Multicolor fluorescent materials offer abundant information carriers and coding capacities, including emission color, intensity, lifetime, and responsiveness. Benefiting from their concealment, uniqueness, and verifiability, these materials can serve as a key bridge in the “human eye-instrument” dual-level anticounterfeiting system.^16,114^

Pyrene derivative with rosin substitution (Py-DAA) demonstrates how external stimuli can independently modulate both molecular structure and aggregate packing to achieve rewritable multicolor fluorescence.^117^ Its fluorescence changed from dark blue to bright green after grinding. Trifluoroacetic acid protonated the imine bonds in Py-DAA and changed its green fluorescence to red (Figure 6b). Therefore, on a paper substrate inked with mixtures of the pristine solid of Py-DAA and other dyes, a lotus flower pattern was drawn to encode visual information. The hidden information—a “rhombus” pattern—was revealed exclusively upon trifluoroacetic acid fuming (Figure 6c). Subsequent exposure to triethylamine vapor caused both the lotus and the rhombus to disappear, restoring the substrate to a blank state resembling a new sheet. Clearly, pyrene-based molecular structures and their aggregated states play a significant role in affecting the fluorescence properties and the practical applications.

Through chemical modification and integration into multicomponent composite systems, spiropyran-based materials have significantly enhanced and diversified the luminescence properties without compromising their inherent photoresponsiveness. This advancement enables a functional evolution from single color switching to dynamic multicolor fluorescence. The dynamic fluorescence variations of the spiropyran-based fluorescent hydrogel under UV irradiation arose from the FRET process (from DEAN-SP to DEAN-MC), accompanied by a color transition from green through yellow to red.^118^ Protonation by H^+^ (DEAN-MCH^+^) suppressed FRET and restored green fluorescence. By assembling organic hydrogel in the pixel array (37 × 8), the encrypted information on “CHN” could be loaded. After UV light irradiation (365 nm), the hidden information presented a dynamic fluorescence change from yellow to red.

Molecular design incorporates merocyanine into the pyrene aromatic system to construct a π-extended conjugated system, yielding the molecule PMC, whose emission and assembly behavior are governed by both an electronic structure and aggregation state.^119^ Under irradiation at 550 nm, PMC underwent photoisomerization to the spiropyran form (PSP) and spontaneously reverted to original PMC in the dark. The substantial polarity difference between PMC and PSP enables efficient regulation of their molecular aggregation behavior by solvent polarity. Varying the ratio of *o*-dichlorobenzene/DMSO mixtures induces pronounced fluorescence chromism in PMC, with the emission shifting from red to orange and then to yellow, whereas PSP consistently exhibits a cyan fluorescence (Figure 7a). The researchers prepared multicolor fluorescent “SEU” patterns on a microplate using mixed solvents with varying ratios. The fluorescent “SEU” letters could be reversibly switched from yellow or orange to cyan fluorescence upon irradiation with different light sources. This photoisomerization-based dynamic optical switching system demonstrates the potential of multicolor fluorescent materials in rewritable information storage and anticounterfeiting applications.

**Figure 7:**
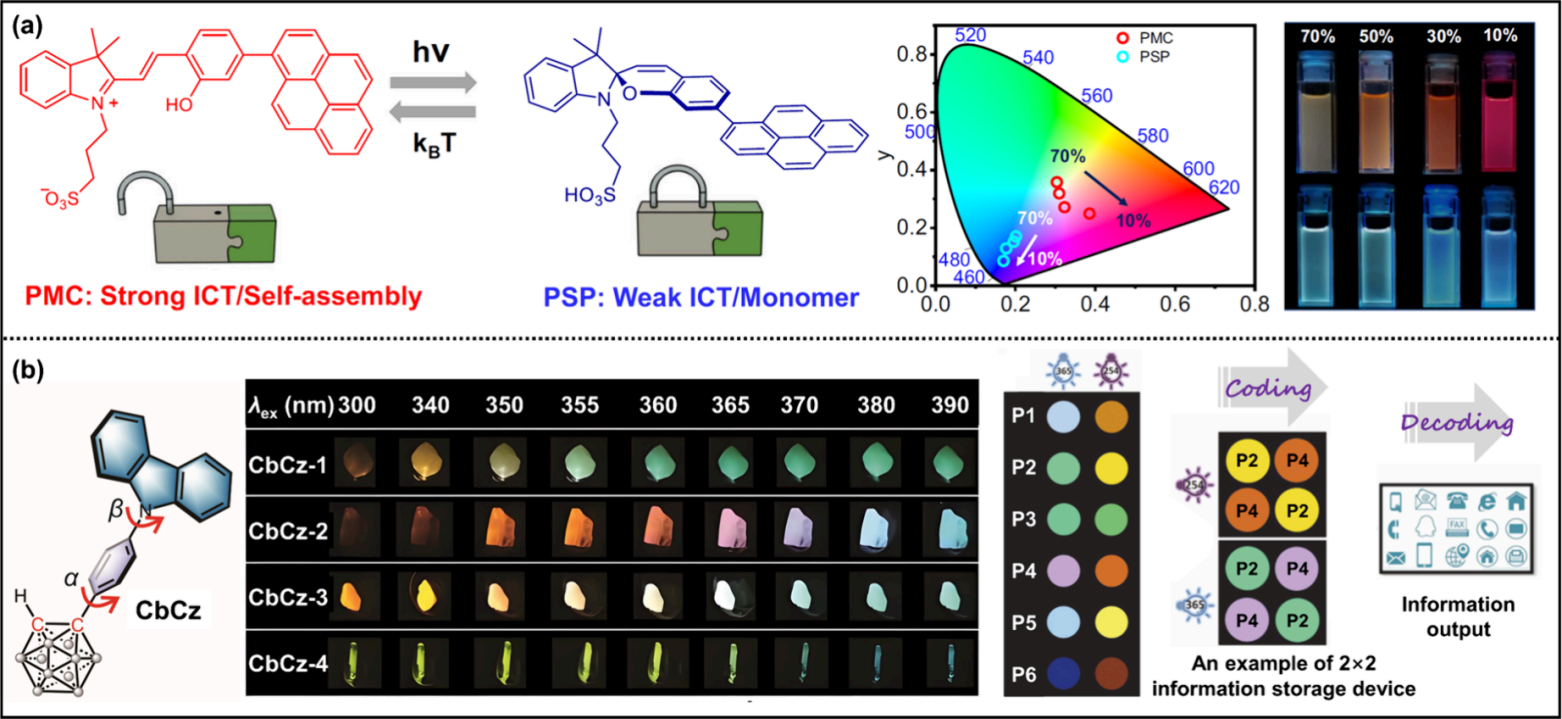
Information storage and encryption. (a) Pyrene-modified merocyanine-based photoswitch with a wide range of photochromic fluorescence in the DMSO/*o*-dichlorobenzene solvent;^119^ (b) structure of CbCz and the fluorescence images of crystals, and their applications in multicolor display and advanced information storage.^122^ Panel (a) reproduced with permission from ref 119. Copyright 2024 Nature Publishing Group. Panel (b) reproduced with permission from ref 122. Copyright 2022 Wiley-VCH.

### 3.3 Information Storage and Encryption

With the rapid development of information technology, efficient information encryption and decryption techniques have attracted great interest from researchers. Fluorescent materials are visible only under ultraviolet light, which is advantageous for information storage and encryption.^109,120,121^

Conformational diversity endows multicolor fluorescence-based anticounterfeiting with a higher level of security. Through rational structural design, a highly versatile fluorescent D-π-A system, denoted as CbCz, was constructed by connecting a carbazole donor and an *o*-carborane acceptor via a conformationally flexible linker.^122^ The deliberate introduction of high rotational freedom represents a key design strategy, fostering remarkable conformational diversity in the crystalline state. By varying the excitation wavelength, the fluorescence emission of the organic crystal can be tuned from cyan/green to orange/red. On this basis, an information storage system was developed using CbCz microcrystals. By employing six distinct CbCz powders and two excitation wavelengths, a 2 × 2 array could generate up to (6 × 2)^4^ = 20,736 unique color combinations (Figure 7b). The employment of additional excitation sources could further expand the available color patterns, underscoring the ultrahigh information density afforded by this material. To demonstrate its dynamic multicolor display capability, a finite-state machine prototype was fabricated using two distinct CbCz crystals. This system exhibited fully reversible fluorescence patterns under 254 nm, 365 nm, or concurrent UV irradiation. The rational flexibility of the D-π-A architecture can effectively expand the number of emission states and enhance color tunability.

Molecular isomerization design integrated with synergistic stimulus-responsive mechanisms achieves an encryption upgrade, converting single-level concealment into dynamic multilevel verification. The excited-state processes of ESIPT-active salicylaldehyde Schiff base skeleton can be finely tuned by varying the substitution position, affording multicolor fluorescence (orange-yellow, blue-green) in the solid state.^123^ Specifically, the salicylaldehyde Schiff base isomer (*p*-TPA-Br) initially exhibited weak yellow fluorescence. Upon exposure to trifluoroacetic acid vapor, the protonation process triggered fluorescence quenching, whereas subsequent treatment with triethylamine vapor restored the fluorescence emission. By combining this property with commercial fluorescent dyes, more complex alphanumeric codes such as “5” and “L, 5, 7” can be encrypted and decrypted. Mechanical grinding enhanced the yellow fluorescence of *p*-TPA-Br, while acid treatment quenched its emission in both the pristine and ground states. Expanding this concept to image encryption (a house pattern), stepwise acid/base treatments progressively revealed hidden features (door/balloon), enabling dynamic multilevel information decryption.

## 4 Challenges and Future Perspectives

Multicolor fluorescent smart materials, as an emerging frontier in functional materials science, demonstrate great promise for both fundamental research and advanced applications. This Outlook summarizes the recent advances in small-organic-molecule-based multicolor fluorescent smart materials, focusing on their design principles, photophysical mechanisms, and application prospects. The strategies for constructing multicolor fluorescent materials include (1) fine-tuning of electronic excited-state processes (ICT, FRET, ESIPT); (2) intrinsic molecular structural transformations (ring-opening/closing, *cis–trans* isomerization, protonation/deprotonation); (3) molecular conformational switching (bent/planar states of DPAC, folding/unfolding of phenothiazine); and (4) modulation of molecular packing modes (H-/J-aggregation, AIE, monomer/excimer, polymorphism). These strategies offer promising avenues for the development of high-performance multicolor fluorescent materials, which have been extensively applied in sensing, anticounterfeiting, information storage, and encryption. Their key advantages lie in their precise, dynamic, and reversible responsive behaviors, as well as high sensitivity favorable for naked-eye visualization.

Despite significant advances in smart fluorescent materials, several critical challenges remain unresolved. First, the fundamental relationship between molecular structure, conformation, packing modes, and photophysical properties is still insufficiently elucidated. Second, there is an urgent demand for smart materials with superior optical properties, including high quantum yield, high contrast, large Stokes shift, as well as exceptional stability and reversibility; notably, integrated systems with multiple optical signal outputs (e.g., fluorescence and circularly polarized luminescence) are still lacking. Third, achieving multiple stimulus responses from a single material and developing switching systems with full-color reproduction remain formidable challenges, particularly for excitation-dependent emissions covering the entire blue-to-red wavelength range. Additionally, practical applications in complex environments impose stringent performance requirements such as high precision, continuity, reversibility, and real-time visualization capabilities.

These challenges simultaneously present unprecedented opportunities for innovation, underscoring the urgent need to provide novel design strategies and develop next-generation smart materials. Molecular derivatization of parent scaffolds and exploration of novel stimulus-responsive moieties (e.g., organic molecules, polymers, and metal–organic frameworks) can be accelerated through machine learning and deep learning approaches. These techniques enable the precise prediction of optical properties and excited-state dynamics, thereby facilitating the rational design of fluorescent molecules with ideal stimulus-responsive photophysical properties. Furthermore, the integration of high-throughput screening with deep-learning algorithms has emerged as a powerful paradigm for elucidating structure–property relationships and optimizing complex optical signal outputs.

To deepen the fundamental understanding of structure–property relationships, further developments of advanced in situ and real-time characterization techniques are required. Ultrafast spectroscopy, in situ microscopy, and time-resolved photoluminescence imaging can dynamically resolve the synergistic mechanisms that govern complex structural evolution (e.g., conformational variation, packing, and assembly/disassembly processes) and excited-state dynamics (e.g., energy/electron transfer and radiative/nonradiative transitions) during stimulus responses. When combined with theoretical modeling, these techniques will provide unprecedented insights into the photophysical mechanisms underlying smart fluorescent materials.

In terms of practical applications, the integration of multicolor smart materials with flexible substrates represents a transformative opportunity for the development of novel wearable sensors and health monitoring devices. The further advancement of multicolor smart fluorescent materials with better biocompatibility (e.g., the NIR-II region) holds great promise for revolutionary breakthroughs in personalized healthcare. Therefore, advancing the field of multicolor fluorescent smart materials critically relies on the rational design of materials that incorporate multiple tunable luminescent centers. To meet practical application requirements, these material systems must concurrently achieve a rapid switching capability, high quantum yield, superior color purity, and robust reversibility. A fundamental and detailed understanding of the structure–property–function relationship is indispensable for guiding this design process, which will ultimately facilitate the creation of high-performance fluorescent materials.
